# Cytokine Gene Expression in Male Genital Lichen Sclerosus: Higher *IL23A* Expression—An Exploratory Study

**DOI:** 10.3390/biomedicines14102374

**Published:** 2026-10-18

**Authors:** Olga Jabłonowska, Zbigniew Jabłonowski, Simona Szkarłat, Radosław Zajdel, Dominika Kulczycka-Wojdala, Agnieszka Żebrowska

**Affiliations:** 1 Department of Dermatology and Venereology, Medical University of Lodz, Hallera 1, 90-647 Lodz, Poland; 2 Department of Urology, Hospital of the Ministry of Interior and Administration in Lodz, 91-425 Lodz, Poland; 3 Department of Economic and Medical Informatics, University of Lodz, 90-214 Lodz, Poland; 4 Central Scientific Laboratory (CoreLab), Medical University of Lodz, 6/8 Mazowiecka Street, 92-215 Lodz, Poland

**Keywords:** male genital lichen sclerosus, IL-23A, IL-17A, JAK/STAT signaling, cytokines, phimosis, quantitative PCR

## Abstract

**Background/Objectives**: Male genital lichen sclerosus (MGLS) is a chronic inflammatory condition that frequently leads to phimosis and other urological complications. The disease can substantially impair quality of life and is associated with an increased risk of malignant transformation. Although several treatment options are available, the immunological mechanisms underlying MGLS remain incompletely understood. This study investigated the expression of *IL22*, *IL23A*, *IL12A*, *IL17A*, *STAT3*, *STAT4*, and *TNF* in MGLS tissue. **Methods**: Tissue samples collected during circumcision from 20 males with histopathologically confirmed MGLS and 17 controls with phimosis but without histopathological evidence of lichen sclerosus were analyzed using real-time quantitative PCR. **Results**: *IL23A* expression was significantly increased in the MGLS group (*p* = 0.0020) and remained significant after Benjamini–Hochberg correction for multiple comparisons (q = 0.0140). Nominal between-group differences in *TNF* and *STAT4* expression were also observed before correction (*p* = 0.0311 and *p* = 0.0331, respectively); however, these findings did not remain statistically significant after correction (q = 0.0772 for both). No statistically significant differences were detected for *IL17A*, *IL22*, *IL12A*, or *STAT3* expression between groups. Interpretation of the *IL17A* result was limited by non-detection in a subset of samples and wide confidence intervals. **Conclusions**: Increased *IL23A* transcript abundance was observed in MGLS tissue, whereas no statistically significant difference in *IL17A* expression was detected. However, the *IL17A* finding should be interpreted cautiously because of limited precision and non-detection in a subset of samples. These findings support further investigation of the biological significance of increased *IL23A* transcript abundance in MGLS.

## 1. Introduction

Male genital lichen sclerosus (MGLS) is a chronic, debilitating dermatosis characterized by a relapsing and remitting course. It is associated with sexual dysfunction, including dyspareunia, erectile difficulties, pruritus, dysuria, and a weakened urinary stream. Lichen sclerosus most commonly affects the anogenital area and, in men, is recognized as a frequent cause of phimosis in adult patients [1,2].

Potent or ultrapotent topical corticosteroids are the first-line treatment for genital lichen sclerosus. Additional therapeutic options include calcineurin inhibitors, surgical procedures, and various off-label therapies. In cases of phimosis, circumcision remains the recommended approach, preferably following an initial course of clobetasol propionate ointment [3]. However, because the immunological pathways involved in the pathogenesis of lichen sclerosus are not fully understood, a significant gap remains in the development of targeted therapeutic approaches for recurrent or postoperative disease.

The prevalence of MGLS has been reported to range between 0.0014% and 0.07% in men, although epidemiological data remain limited and the condition is likely underdiagnosed. The most common age at diagnosis is between the third and fifth decades of life [4,5,6]. The pathogenesis of lichen sclerosus is believed to be influenced by multiple factors, including genetic predisposition, infections, environmental triggers, and autoimmune mechanisms. Chronic inflammation and oxidative stress are also thought to play important roles in disease development [3].

Immunological pathways involved in lichen sclerosus remain under active investigation, with the aim of identifying potential therapeutic targets. Early immunological studies suggested a predominance of a Th1-mediated immune response, as demonstrated by increased tissue expression of interferon-γ (IFN-γ), tumor necrosis factor-α (TNF-α), and interleukin-1α (IL-1α) [7]. Subsequent studies further supported the predominance of Th1-related immune responses [8,9]. In contrast, the involvement of IL-17 remains controversial: increased IL-17 expression was reported by Baran et al. in vulvar lichen sclerosus, whereas Terlou et al. did not observe elevated IL-17 levels [9,10].

More recently, single-cell and spatial transcriptomic analyses by Sun et al. demonstrated activation of interferon-related signaling pathways, including increased JAK/STAT pathway activity, in vulvar lichen sclerosus lesions [8]. In addition, a recent exploratory NanoString-based gene-expression profiling study by Poffet et al. identified broad alterations in inflammation-related genes and significant upregulation of IL-12-mediated and IL-12/STAT4-associated signaling in LS compared with non-diseased controls [11]. Taken together, these findings suggest that cytokine networks associated with JAK/STAT signaling may be involved in lichen sclerosus, although direct evidence from male genital tissue remains underexplored. Moreover, a recent exploratory pilot study reported associations between IL-17/IL-23-related signaling and clinical features of vulvar lichen sclerosus, further supporting the potential relevance of these pathways in disease pathogenesis [12]. However, targeted evaluation of *IL23A*, *IL17A*, *IL22*, *IL12A*, *STAT3*, *STAT4*, and *TNF* expression in a histopathologically defined MGLS cohort remains limited, and available data on IL-17 expression remain conflicting.

Therefore, the aim of the present study was to analyze the expression of selected inflammatory markers—*IL22*, *IL23A*, *IL12A*, *IL17A*, *STAT3*, *STAT4*, and *TNF*—in tissue obtained from patients with phimosis who underwent circumcision and had a histopathologically confirmed diagnosis of MGLS.

## 2. Materials and Methods

### 2.1. Study Design and Participants

This was a single-center, cross-sectional observational study conducted between September 2021 and December 2023. During this period, 43 adult male patients with phimosis who were eligible for circumcision were considered for participation in the study. Six patients were not included in the final analysis: one because consent for participation was not provided, three because tissue samples were unsuitable for molecular analysis owing to storage or transport problems, and two because predefined exclusion criteria were met. The final gene-expression analysis included 37 participants: 20 patients with histopathologically confirmed MGLS and 17 controls with phimosis but without histopathological evidence of lichen sclerosus. Demographic and clinical data, including age and previous topical treatment, were collected.

All participants included in the final analysis underwent circumcision because of phimosis. Patients with active infections, malignant disease, systemic immunosuppression, psoriasis, or other known inflammatory dermatoses at the time of recruitment were excluded. Previous treatment with topical corticosteroids or topical calcineurin inhibitors was recorded. Participants had not used either treatment for at least four weeks before circumcision. Treatment history was unavailable for a minority of participants.

The seven target genes were selected a priori based on their involvement in inflammatory pathways potentially relevant to lichen sclerosus. The panel included genes related to IL-23/Th17-associated responses (*IL23A*, *IL17A*, and *IL22*), IL-12/Th1-associated signaling (*IL12A* and *STAT4*), *STAT3* as a key transcription factor involved in IL-23/Th17 signaling, and *TNF* as a broader pro-inflammatory mediator. No formal a priori sample-size calculation was performed because of the exploratory nature of the study and the limited availability of eligible circumcision specimens.

### 2.2. Histopathological Assessment

Foreskin specimens obtained during circumcision were submitted for routine histopathological examination. All specimens were evaluated by a specialist pathologist who was unaware of the qPCR results, and the diagnosis was routinely reviewed and confirmed by a second pathologist. The diagnosis of lichen sclerosus was based on the overall histopathological pattern, including characteristic features such as epidermal architectural changes with hyperkeratosis and/or epidermal thinning, basal vacuolar/interface degeneration, homogenization or sclerosis of the superficial dermis, and chronic lymphocytic inflammatory infiltrates. Participants were assigned to the MGLS group when lichen sclerosus was confirmed histopathologically and to the control group when phimosis was present without histopathological evidence of lichen sclerosus. No formal histopathological severity score or staging system was applied. Foreskin tissue obtained during circumcision was divided into separate portions for routine histopathological examination and molecular analysis; therefore, the exact spatial correspondence between the tissue examined histologically and the tissue used for RNA analysis could not be ensured.

### 2.3. Tissue Collection and RNA Extraction

Tissue samples obtained during circumcision were immediately preserved in RNAlater solution (Sigma-Aldrich, St. Louis, MO, USA) and stored frozen until RNA extraction. Total RNA was extracted from 10 mg of foreskin tissue using the RNeasy Mini Kit (Qiagen, Hilden, Germany) according to the manufacturer’s instructions. Tissue samples were initially cut into small fragments using a sterile scalpel on a Petri dish. The fragmented tissue was transferred to microcentrifuge tubes containing RLT lysis buffer supplemented with β-mercaptoethanol and subsequently homogenized using a TissueRuptor homogenizer (Qiagen, Hombrechtikon, Switzerland). The remaining RNA extraction steps were performed in accordance with the manufacturer’s protocol.

RNA concentration was measured using a Qubit 4 Fluorometer with the Qubit RNA High Sensitivity Assay Kit (Thermo Fisher Scientific, Eugene, OR, USA).

### 2.4. Reverse Transcription and Quantitative PCR Analysis

Reverse transcription was performed using the High-Capacity cDNA Reverse Transcription Kit (Thermo Fisher Scientific, Vilnius, Lithuania). A total of 2000 ng of RNA was used as input for each 20 μL reverse-transcription reaction. The reaction mixture contained 2 μL of 10× RT buffer, 0.8 μL of 25× dNTP mix (100 mM), 2 μL of 10× RT random primers, 1 μL of MultiScribe Reverse Transcriptase, and 1 μL of RNase inhibitor, together with RNA and nuclease-free water to a final volume of 20 μL.

Reverse transcription was performed at 25 °C for 10 min, 37 °C for 120 min, and 85 °C for 5 min. The resulting cDNA samples were stored at −20 °C until further analysis.

Quantitative PCR was performed using TaqMan Gene Expression Assays (Thermo Fisher Scientific, Pleasanton, CA, USA) for *IL12A* (Hs01073447_m1), *IL17A* (Hs00174383_m1), *IL22* (Hs01574154_m1), *IL23A* (Hs00372324_m1), *STAT3* (Hs00374280_m1), *STAT4* (Hs01028017_m1), *TNF* (Hs01113624_g1), and *ACTB* (Hs99999903_m1). *ACTB* was used as the endogenous reference gene.

Each qPCR reaction was performed in a total volume of 10 μL containing 50 ng of cDNA, 5 μL of TaqMan Fast Advanced Master Mix (Thermo Fisher Scientific, Vilnius, Lithuania) and 0.5 μL of the appropriate TaqMan Gene Expression Assay. Reactions were performed in technical duplicate using a 7900HT Fast Real-Time PCR System (Applied Biosystems, Foster City, CA, USA).

The thermal cycling protocol consisted of an initial activation step at 95 °C for 20 s, followed by 40 cycles of denaturation at 95 °C for 1 s and annealing/extension at 60 °C for 20 s.

Data were analyzed using SDS Software version 2.4 and DataAssist Software version 3.01 (Applied Biosystems, Foster City, CA, USA). For each sample, ΔCt was calculated as the difference between the mean Ct value of the target gene and the mean Ct value of *ACTB*. Sample 17 was used as the calibrator for ΔΔCt calculations, and relative quantity values were calculated using the 2^−ΔΔCt^ method. The calibrator was used only to define the scale of relative quantity values used for graphical presentation; inferential between-group comparisons were based on ΔCt values and were therefore independent of the choice of calibrator. In DataAssist Software, the maximum allowable Ct value was set at 40.0. Raw “Undetermined” wells were assigned Ct = 40 by the software; this value represented an operational software ceiling rather than a validated limit of detection or quantification.

### 2.5. Statistical Analysis

Statistical analyses were performed using StatsDirect version 4.0.4 (StatsDirect Ltd., Wirral, UK). Between-group comparisons of gene expression were based on ΔCt values. Normality was assessed using the Shapiro–Wilk test. Variables with approximately normal distributions in both groups were compared using the unpaired Student’s *t*-test, whereas variables that did not meet the normality assumption in at least one group were analyzed using the Mann–Whitney U test. Effect sizes were reported as Hedges’ g or rank-biserial correlation (r_rb), as appropriate. *p* values for the seven analyzed genes were adjusted using the Benjamini–Hochberg false discovery rate procedure. All tests were two-sided, and q < 0.05 was considered statistically significant. Relative quantity values were used only for graphical presentation.

As a sensitivity analysis, −ΔCt values for each gene were modeled using age-adjusted linear regression, with diagnostic group and continuous age included as predictors. The group coefficient was expressed as an approximate fold change (MGLS/control). Heteroskedasticity-consistent HC3 standard errors were used, and *p* values were adjusted across the seven genes using the Benjamini–Hochberg procedure. The age-adjusted analysis was also repeated after excluding participants with unavailable treatment history. For *IL17A*, the minimum detectable standardized difference was calculated assuming 80% power and a two-sided α = 0.05.

An exploratory age-adjusted reference-gene sensitivity analysis was performed in a subset of 16 participants with available *GUSB* measurements (nine MGLS and seven controls). Target-gene expression was normalized using *ACTB* alone, *GUSB* alone, or the mean Ct of *ACTB* and *GUSB* as a dual reference.

These additional analyses were performed using Python 3.13.5 with NumPy 2.3.5, SciPy 1.17.0, and statsmodels 0.14.6.

### 2.6. Ethical Approval

The study protocol was approved by the Bioethics Committee of the Medical University of Lodz (No. RNN/421/19/KE). Written informed consent was obtained from all study participants.

## 3. Results

### 3.1. Study Population

The study included 20 male patients with histopathologically confirmed MGLS and 17 controls with phimosis but without histopathological evidence of lichen sclerosus. The mean age was 56.6 ± 18.4 years in the MGLS group and 47.1 ± 20.2 years in the control group. There was no statistically significant difference in age between the groups (unpaired Student’s *t*-test, *p* = 0.144). In the MGLS group, 13 patients reported no previous treatment, two had previously used topical corticosteroids, and treatment history was unavailable for five patients. In the control group, 13 participants reported no previous treatment, two had previously used topical corticosteroids, one had previously used a topical calcineurin inhibitor, and treatment history was unavailable for one participant. No participant reported previous treatment with photodynamic therapy, laser therapy, or platelet-rich plasma in the study questionnaire. The demographic and clinical characteristics of the study population are summarized in Table 1.

Histopathological findings in the 17 control specimens were heterogeneous. Six specimens showed no histopathological evidence of lichen sclerosus without a dominant inflammatory pattern, five showed predominantly hyperkeratotic changes, one showed parakeratosis, two showed koilocytic changes suggestive of HPV infection, and two showed chronic inflammatory infiltrates. One specimen showed acanthosis, parakeratosis, hyperkeratosis, and a relatively intense chronic inflammatory infiltrate, with the overall histopathological appearance considered most consistent with lichen planus. None of the control specimens was diagnosed as lichen sclerosus.

### 3.2. Gene-Expression Analysis

**Figure 1:**
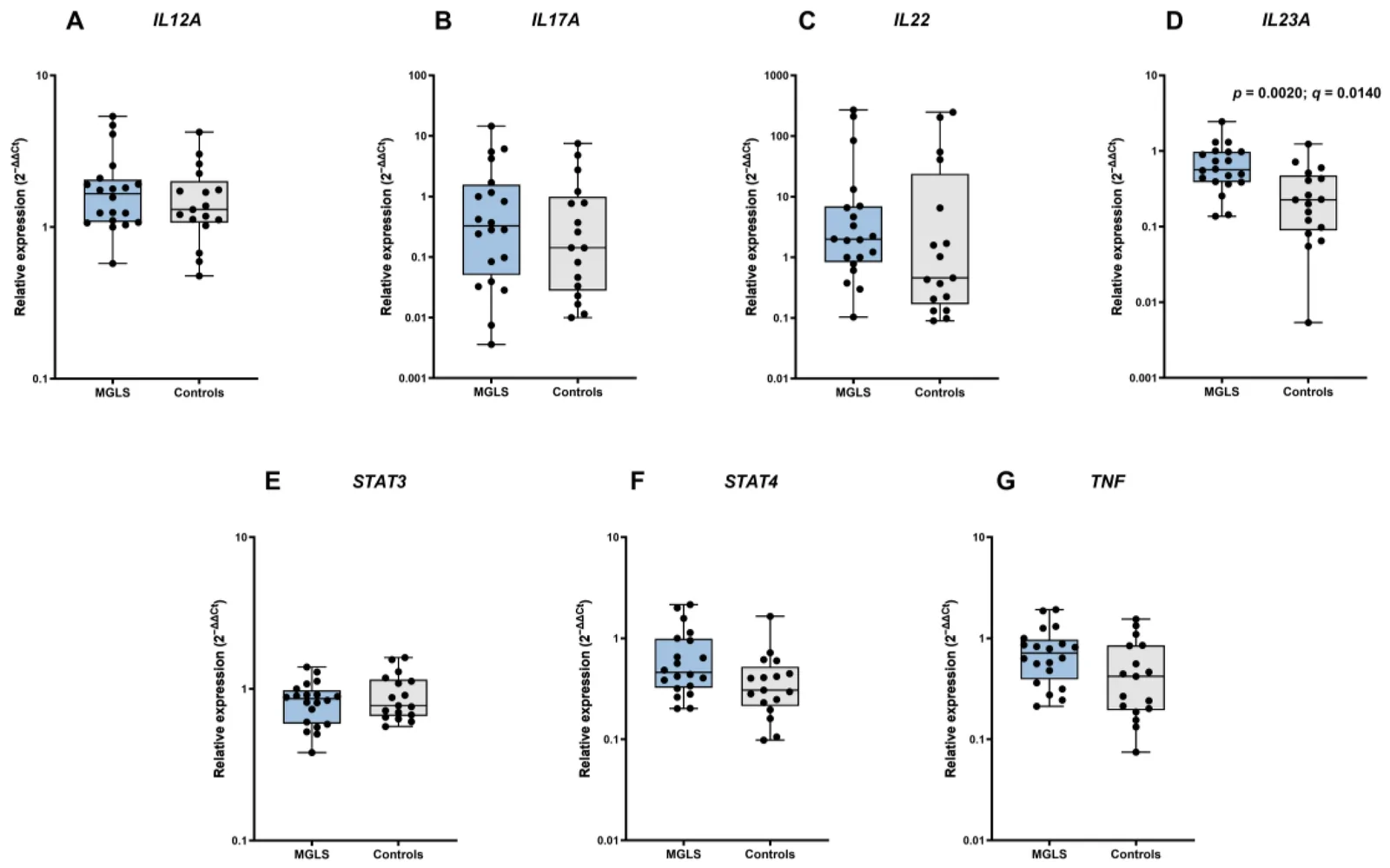
Relative mRNA expression of *IL12A* (**A**), *IL17A* (**B**), *IL22* (**C**), *IL23A* (**D**), *STAT3* (**E**), *STAT4* (**F**), and *TNF* (**G**) in patients with male genital lichen sclerosus (MGLS; *n* = 20) and controls (*n* = 17). Relative quantity (RQ) values are shown. Boxes represent the interquartile range, horizontal lines indicate medians, and whiskers indicate the minimum and maximum values.

*IL23A* expression was higher in tissue samples from patients with MGLS than in controls (Mann–Whitney U test, *p* = 0.0020; rank-biserial correlation = 0.58) and remained significant after Benjamini–Hochberg correction for multiple comparisons (q = 0.0140). Nominal between-group differences in *TNF* and *STAT4* expression were observed before correction (*p* = 0.0311 and *p* = 0.0331, respectively); however, these differences did not remain statistically significant after correction (q = 0.0772 for both). No statistically significant differences were detected in *IL17A*, *IL22*, *IL12A*, or *STAT3* expression between the study groups (all unadjusted *p* > 0.05). Descriptive statistics, effect sizes, unadjusted *p* values, and Benjamini–Hochberg-adjusted q values are presented in Table 2, whereas relative expression values are shown in Figure 1.

For *IL17A*, at least one technical well was undetermined in 3/20 MGLS samples and 5/17 control samples, while all technical wells were undetermined in two participants in each group.

In an additional age-adjusted sensitivity analysis, *IL23A* expression remained higher in the MGLS group (fold change 2.99, 95% CI 1.41–6.35; *p* = 0.0055; q = 0.0388). No other gene remained statistically significant after Benjamini–Hochberg correction. After excluding participants with unavailable treatment history, *IL23A* expression remained higher in the MGLS group (fold change 3.23, 95% CI 1.46–7.12; *p* = 0.0052; q = 0.0363).

In the subset with available *GUSB* measurements, the direction of the age-adjusted *IL23A* association was consistent when expression was normalized using *ACTB* alone, *GUSB* alone, or the mean Ct of *ACTB* and *GUSB* as a dual reference. None of these subset analyses remained statistically significant after Benjamini–Hochberg correction for multiple comparisons.

## 4. Discussion

Our findings revealed increased *IL23A* expression in the MGLS group, whereas nominal between-group differences were observed for *STAT4* and *TNF* mRNA expression. After correction for multiple comparisons, only the difference in *IL23A* expression remained statistically significant, whereas the findings for *STAT4* and *TNF* did not. The findings for *STAT4* and *TNF* should be considered exploratory. Clinical responses to JAK inhibitors and TNF-α-targeted therapies have been described in case reports and a systematic review, providing clinical context for further research in lichen sclerosus [13,14,15,16].

Beyond pharmacological approaches, physical and energy-based modalities such as photodynamic therapy have also been explored in MGLS, although current clinical evidence remains limited [17,18,19]. More broadly, tissue-level studies of energy-based interventions illustrate the potential value of histological and immunohistochemical approaches for characterizing local biological responses to such treatments [20]. Similar mechanistic studies in MGLS could help clarify whether these modalities modulate disease-relevant tissue responses.

No statistically significant difference in *IL17A* expression was detected between the study and control groups. IL-23 plays an important role in the regulation of inflammatory responses by promoting the expansion and maintenance of Th17 cells, a major source of IL-17 [21]. The IL-23/IL-17 axis is recognized as a central pathogenic pathway in several chronic inflammatory skin diseases, including psoriasis and hidradenitis suppurativa, and the therapeutic blockade of IL-23 or IL-17 has demonstrated substantial clinical efficacy in these conditions [22,23,24]. However, increased IL-23 expression does not necessarily correspond to increased IL-17 expression. IL-23 is considered to play a greater role in the stabilization and maintenance of pathogenic Th17 responses than in the initial induction of IL-17 production [25]. In addition, IL-17 expression is influenced by the local cytokine environment and other regulatory factors, including IFN-γ and IL-12 [26]. Furthermore, accumulating evidence suggests that IL-17A and IL-17F production may occur independently of IL-23 signaling in certain tissues and inflammatory settings, further underscoring the complexity of IL-23-associated immune responses [27]. Taken together, these mechanisms provide biological context for the observed pattern of higher *IL23A* transcript abundance alongside a non-significant *IL17A* comparison; however, the present study cannot establish an IL-17-independent mechanism.

Recently, an exploratory pilot study in vulvar lichen sclerosus reported that IL-23 expression correlated with disease duration, whereas IL-17 expression was associated with atrophic changes [12]. However, data regarding IL-17 expression in lichen sclerosus remain conflicting. One study of vulvar lichen sclerosus reported increased IL-17 expression, whereas another found no significant difference in IL-17 levels [9,10]. These discrepancies may reflect differences in study populations, as the available data on IL-17 expression in lichen sclerosus largely derive from studies conducted in female cohorts, whereas our study included only male patients. Moreover, differences in the local immune microenvironment and tissue-resident immune cell composition between female and male genital tissues may contribute to distinct cytokine expression patterns [28].

Overall, these observations highlight the complexity of IL-23-related biology and suggest that increased *IL23A* transcript abundance in MGLS may not necessarily be accompanied by increased *IL17A* expression.

Previous transcriptomic studies have demonstrated activation of JAK/STAT-associated pathways in lichen sclerosus [8]. In addition, Poffet et al. reported activation of IL-12/STAT4-associated signaling in a small molecular analysis comprising four LS samples [11]. Our analysis showed nominally higher *STAT4* expression in the MGLS group compared with controls; however, this difference did not remain statistically significant after correction for multiple comparisons. STAT4 is a key transcription factor involved in IL-12-mediated Th1-cell differentiation and IFN-γ production [29]. Lee et al. demonstrated that IL-23, although less potently than IL-12, can also activate STAT4 and contribute to the functional reprogramming of Th17 cells [30]. This finding provides a biological context for the nominally higher *STAT4* expression observed in our study; however, our transcript-level data do not establish functional IL-23–STAT4 signaling or activation of the IL-23 pathway.

Tumor necrosis factor-α (TNF-α) is a pleiotropic pro-inflammatory cytokine involved in immune regulation, and its dysregulation has been implicated in the pathogenesis of chronic inflammatory and autoimmune diseases [31]. Increased TNF-α expression has been reported in several inflammatory skin diseases, including psoriasis, hidradenitis suppurativa, and lichen planus, supporting its role as a mediator of cutaneous inflammation [32,33]. Previous immunohistochemical studies demonstrated enhanced TNF-α staining in lichen sclerosus tissue, particularly within dermal inflammatory infiltrates, with less pronounced staining observed in the sclerotic zone [7]. In our cohort, *TNF* mRNA expression was higher in patients with MGLS than in controls. However, this difference did not remain statistically significant after correction for multiple comparisons. Accordingly, the result should be regarded as exploratory, although it may be consistent with previous observations suggesting the involvement of TNF-α in the pathogenesis of lichen sclerosus.

The main limitations of this study include the relatively small sample size, the single-center design, and the lack of protein-level validation of the gene-expression findings. The relatively small sample size increases the risk of Type II error for the non-significant gene-expression comparisons. For *IL17A*, the minimum detectable standardized difference at 80% power and a two-sided α = 0.05 was d = 0.951; therefore, the non-significant result should not be interpreted as evidence of absent biological involvement. The approximately 10-year difference in mean age between groups represents a potential source of confounding; although the age-adjusted sensitivity analysis yielded a similar *IL23A* association, residual confounding cannot be completely excluded. Moreover, mRNA abundance does not directly reflect cytokine protein concentrations or pathway activation. In addition, IL-23 is a heterodimer composed of the p19 subunit encoded by *IL23A* and the p40 subunit encoded by *IL12B*. Because *IL12B* expression and IL-23 protein were not measured, increased *IL23A* transcript abundance cannot be interpreted as evidence of increased production or activity of functional IL-23. Another limitation is the use of *ACTB* as the sole reference gene in the primary analysis. An exploratory age-adjusted sensitivity analysis using *GUSB* measurements available for a subset of participants showed a consistent direction of the *IL23A* association across alternative normalization approaches; however, the limited subset size precluded formal validation of reference-gene stability. Additionally, the control group consisted of patients with non-LS phimosis rather than healthy individuals, which may have influenced the observed gene-expression profiles. Although all control specimens lacked a histopathological diagnosis of lichen sclerosus, early or focal LS may show subtle or nonspecific changes. Because the tissue portions used for histopathological and molecular analyses were not spatially identical, some degree of histopathological misclassification cannot be completely excluded. Histopathological severity or stage was not systematically assessed, and reliable data on symptom duration were not available for all participants; therefore, associations between disease chronicity or severity and gene-expression patterns could not be evaluated. Some patients had also received topical treatment before enrollment. Although a 4-week washout period for topical therapies was implemented before tissue collection, the potential long-term effects of previous treatment on gene expression cannot be entirely excluded. In addition, treatment history was unavailable for a greater proportion of participants in the MGLS group than in the control group; although the exclusion of participants with unavailable treatment history yielded a similar *IL23A* association, and residual bias related to incompletely documented prior treatment cannot be excluded. Accordingly, the results should be interpreted in light of these limitations. Further studies are warranted to better characterize the immunological pathways involved in MGLS.

## 5. Conclusions

The present findings provide evidence of altered inflammatory gene expression in MGLS. Increased *IL23A* expression was observed in MGLS tissue, whereas no statistically significant difference in *IL17A* expression was detected between groups. These findings identify increased *IL23A* transcript abundance as a potential molecular feature of MGLS and support further investigation of its biological significance. Nominal between-group differences in *TNF* and *STAT4* expression were also observed; however, these findings did not remain statistically significant after correction for multiple comparisons and should therefore be interpreted cautiously. Taken together with previous studies, these exploratory findings warrant further investigation of TNF-α-related inflammation and JAK–STAT-associated signaling in MGLS.

## Figures and Tables

**Table 1:** Demographic and clinical characteristics of the study population.

Characteristic	MGLS (*n* = 20)	Controls (*n* = 17)
Number of participants	20	17
Age, years (mean ± SD)	56.6 ± 18.4	47.1 ± 20.2
Previous topical corticosteroid treatment, *n* (%)	2 (10.0)	2 (11.8)
Previous topical calcineurin inhibitor treatment, *n* (%)	0 (0.0)	1 (5.9)
No previous treatment, *n* (%)	13 (65.0)	13 (76.5)
Treatment history unavailable, *n* (%)	5 (25.0)	1 (5.9)

Data are presented as mean ± SD or number (%). MGLS, male genital lichen sclerosus; SD, standard deviation.

**Table 2:** Comparison of ΔCt values for the investigated genes between patients with male genital lichen sclerosus and controls.

Gene	MGLS (*n* = 20)	Controls (*n* = 17)	Statistical Test	Effect Size	Unadjusted *p* Value	BH-Adjusted q Value
*IL12A*	11.65 ± 0.81	11.91 ± 0.83	Unpaired Student’s *t*-test	Hedges’ g = −0.32	0.330	0.448
*IL17A*	16.21 ± 3.27	16.90 ± 3.06	Unpaired Student’s *t*-test	Hedges’ g = −0.21	0.514	0.514
*IL22*	16.51 (14.73–17.68)	18.63 (14.79–19.79)	Mann–Whitney U test	r_rb = 0.29	0.133	0.232
*IL23A*	14.28 (13.49–14.81)	15.60 (14.66–16.81)	Mann–Whitney U test	r_rb = 0.58	0.002	0.014
*STAT3*	4.25 ± 0.48	4.11 ± 0.48	Unpaired Student’s *t*-test	Hedges’ g = 0.28	0.384	0.448
*STAT4*	7.40 ± 1.03	8.15 ± 1.02	Unpaired Student’s *t*-test	Hedges’ g = −0.72	0.033	0.077
*TNF*	9.95 ± 0.92	10.76 ± 1.27	Unpaired Student’s *t*-test	Hedges’ g = −0.72	0.031	0.077

Data are presented as mean ± standard deviation or median (Q1–Q3), as appropriate. Statistical analyses and effect sizes were based on ΔCt values. *p* values were adjusted for seven comparisons using the Benjamini–Hochberg procedure. BH, Benjamini–Hochberg; MGLS, male genital lichen sclerosus; Q1, first quartile; Q3, third quartile.

## Data Availability

The data supporting the findings of this study are available from the corresponding author upon reasonable request. The data are not publicly available due to privacy and ethical considerations.

## Abbreviations

The following abbreviations are used in this manuscript:

LS	Lichen sclerosus
MGLS	Male genital lichen sclerosus
